# Transition from antigenemia to quantitative nucleic acid amplification testing in cytomegalovirus-seropositive kidney transplant recipients receiving preemptive therapy for cytomegalovirus infection

**DOI:** 10.1038/s41598-022-16847-3

**Published:** 2022-07-27

**Authors:** Mônica Rika Nakamura, Lúcio R. Requião-Moura, Roberto Mayer Gallo, Camila Botelho, Júlia Taddeo, Laila Almeida Viana, Cláudia Rosso Felipe, José Medina-Pestana, Hélio Tedesco-Silva

**Affiliations:** 1 Hospital do Rim, Fundação Oswaldo Ramos, São Paulo, Brazil; 2 Nephrology Division, Department of Medicine, Universidade Federal de São Paulo, São Paulo, Brazil

**Keywords:** Nephrology, Infectious diseases

## Abstract

Due to the high costs, the strategy to reduce the impact of cytomegalovirus (CMV) after kidney transplant (KT) involves preemptive treatment in low and middle-income countries. Thus, this retrospective cohort study compared the performance of antigenemia transitioned to quantitative nucleic acid amplification testing, RT-PCR, in CMV-seropositive KT recipients receiving preemptive treatment as a strategy to prevent CMV infection. Between 2016 and 2018, 363 patients were enrolled and received preemptive treatment based on antigenemia (n = 177) or RT-PCR (n = 186). The primary outcome was CMV disease. Secondarily, the CMV-related events were composed of CMV-infection and disease, which occurred first. There were no differences in 1-year cumulative incidence of CMV-disease (23.7% vs. 19.1%, p = 0.41), CMV-related events (50.8% vs. 44.1%, p = 0.20), neither in time to diagnosis (47.0 vs. 47.0 days) among patients conducted by antigenemia vs. RT-PCR, respectively. The length of CMV first treatment was longer with RT-PCR (20.0 vs. 27.5 days, p < 0.001), while the rate of retreatment was not different (14.7% vs. 11.8%, p = 0.48). In the Cox regression, acute rejection within 30 days was associated with an increased the risk (HR = 2.34; 95% CI = 1.12–4.89; p = 0.024), while each increase of 1 mL/min/1.73 m^2^ of 30-day eGFR was associated with a 2% reduction risk of CMV-disease (HR = 0.98; 95% CI = 0.97–0.99; p = 0.001). In conclusion, acute rejection and glomerular filtration rate are risk factors for CMV disease, showing comparable performance in the impact of CMV-related events between antigenemia and RT-PCR for preemptive treatment.

## Introduction

The cytomegalovirus (CMV) infection is one of the most common infectious events after solid organ transplantation, affecting 20 to 60% of kidney transplant recipients^1–3^, increasing morbidity, costs and leading to a possible negative impact on graft survival^3^. The effects of the CMV infection have been traditionally characterized as direct and indirect^4^. Although the indirect effects have been questionable recently, the direct effects, such as symptoms and laboratory changes attributable to CMV and the invasive disease, are still a field of concern after kidney transplantation^4,5^. Cytomegalovirus replication occurs mainly in the first 3 months after the transplant, and the clinical presentation is now well defined according to international guidelines in infection, disease, and invasive disease^4,6^.

Considering the latent CMV infection is widely detected among candidates for kidney engraftment^7^, the risk of CMV active infection after transplantation should be evaluated, and a strategy to reduce the impact of direct effect has to be adopted^4^. Currently, there are two efficacy and safe alternatives for preventing outcomes related to CMV after transplantation: universal pharmacological prophylaxis or preemptive treatment^8–10^. Although universal prophylaxis seems to be associated with lower CMV-related effects, some disadvantages have been highlighted: toxicity, late-onset CMV disease, risk of resistance, and costs^4^. In Brazil, for instance, prophylaxis with oral valganciclovir for 3 months can cost 3 to 7 times more than the preemptive treatment, depending on graft function and frequency of monitoring^11^. Thus, due to the high cost, the way to reduce the impact of CMV involves targeted prevention through preemptive treatment, especially in low and middle-income countries.

For preemptive treatment, patients must be strictly monitored for CMV replication throughout a laboratory method to detect viral load. For many years, many services performed a semi-quantitative test, an immunofluorescence assay based on monoclonal antibodies that detect the viral antigen, such as the pp65 antigenemia^12–14^. However, in the last two decades, it has been replaced by quantitative nucleic acid testing, especially by standardization ultra-sensitivity real-time polymerase chain reaction (RT-PCR), such that it is currently the preferred method for CMV management^4^. In 2017, we started implementing standardized RT-PCR for the preemptive treatment in our center, replacing the antigenemia completely 1 year later. This change in the clinical routine designed a natural experiment with the potential to measure CMV-related events as outcomes in two different eras. Therefore, in the present study, we aimed to compare the performance of antigenemia transitioned to quantitative nucleic acid amplification testing, RT-PCR, in CMV-seropositive kidney transplant recipients receiving preemptive treatment and to evaluate the potential clinical predictors of the CMV-related events.

## Results

**Figure 1:**
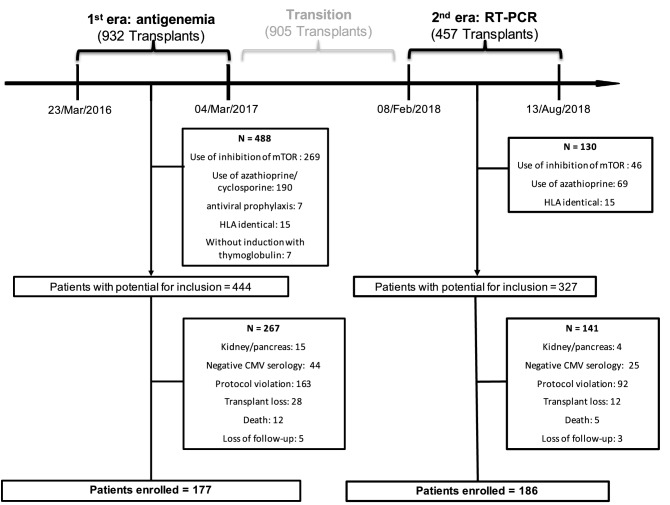
Flowchart of the population. The transition period is from March 2017 to February 2018, when the service had adopted both methods for viremia detection. CMV, cytomegalovirus; HLA, human leukocyte antigen; mTOR, mammalian target of rapamycin; RT-PCR, real-time polymerase chain reaction.

Between March 2016 and August 2018, a total of 2294 kidney transplants were performed in our center. Initially, 905 patients were excluded because they were transplanted in a transition period (from March 2017 to February 2018). In the antigenemia era (March 2016 to March 2017), 932 patients had been transplanted; however, 488 recipients did not present inclusion criteria, and 267 had exclusion criteria, as depicted in Fig. 1. On the other hand, in the RT-PCR era (February 2018 to August 2018), 457 patients had been transplanted; however, 130 recipients did not present inclusion criteria, and 141 had exclusion criteria (Fig. 1). Among patients excluded due to death or graft loss (n = 65), 38 had died or had graft loss within 60 days of transplantation. All 17 deaths were verified, and no one was attributed to the CMV event. Therefore, 177 patients were enrolled for the antigenemia era, whereas 186 were for the RT-PCR era.

### Demography data according to testing era: antigenemia and RT-PCR

Demographic data are shown in Table 1. Patients were 49.0 years old, 54.8% males and 54.8% whites. The etiology for chronic kidney disease was unknown for 44.6%, and 93.7% had undergone hemodialysis as a renal replacement treatment before transplantation; only 12.7% of patients had been submitted to a retransplantation. Donors were 52.0 years old, 52.9% male, and 52.1% white. Most transplants were performed with a deceased donor (96.1%), whose median KDPI value was 80.0. The cold ischemia time was 23.1 h, and the delayed graft function (DGF) occurred in 187 patients (51.5%).

**Table 1:** Demographic data according to the era: antigenemia and PCR.

Results	Total (N = 363)	Antigenemia era (N = 177)	PCR era (N = 186)	P-value
Recipient age*,* years	49.0 (37.0; 57.0)	50.0 (39.2; 57.0)	50.0 (41.0; 60.0)	0.38
Recipient sex, *male,* N (%)	199 (54.8)	92 (52.0)	107 (57.5)	0.29
**Recipient ethnicity, N (%)**				0.007
White	199 (54.8)	83 (46.9)	116 (62.4)	
Pardo	177 (32.3)	71 (40.1)	46 (24.7)	
Afro Brazilian	40 (11.0)	21 (11.9)	19 (10.2)	
Other	7 (1.9)	2 (1.1)	5 (2.7)	
**Cause of CKD, N (%)**				0.22
Unknown	162 (44.6)	80 (45.2)	82 (44.1)	
Diabetes mellitus	52 (14.3)	22 (12.4)	30 (16.1)	
Glomerulonephritis	47 (12.9)	18 (10.2)	29 (15.6)	
Hypertension	29 (8.0)	18 (10.2)	11 (5.9)	
ADPKD	23 (6.3)	10 (5.6)	13 (7.0)	
Other	50 (13.8)	29 (16.4)	21 (11.3)	
Time on dialysis, *months*	42.0 (20.0; 79.0)	56.0 (31.2; 88.0)	42.0 (22.0; 88.0)	0.03
Type of dialysis, *hemodialysis* N (%)	340 (93.7)	162 (91.5)	178 (95.7)	0.10
Retransplant, N (%)	46 (12.7)	24 (13.6)	22 (11.8)	0.62
**PRA Class I, N (%)**				0.01
0–29%	273 (75.2)	123 (69.5)	150 (80.6)	
30–80%	56 (15.4)	30 (16.9)	26 (14.0)	
> 80%	34 (9.4)	24 (13.6)	10 (5.4)	
**PRA Class II, N (%)**				0.03
0–29%	315 (86.8)	148 (83.6)	167 (89.8)	
30–80%	29 (8.0)	21 (11.9)	8 (4.3)	
> 80%	19 (5.2)	8 (4.5)	11 (5.9)	
Donor type, deceased, N (%)	349 (96.1)	170 (96.0)	179 (96.2)	0.92
Donor age, years	52.0 (42.0; 60.0)	53.0 (42.0; 62.0)	52.0 (42.0; 58.0)	0.003
Donor sex, *male,* N (%)	192 (52.9)	85 (48.0)	107 (57.5)	0.07
**Donor ethnicity, N (%)**				0.28
White	189 (52.1)	93 (52.5)	96 (51.6)	
Pardo	139 (38.3)	64 (36.2)	75 (40.3)	
Afro Brazilian	32 (8.8)	17 (9.6)	15 (8.1)	
Other	3 (0.8)	3 (1.7)	–	
Donor CMV-IgG, *positive*, N (%)^a^	290 (91.8)	143 (89.9)	147 (93.6)	0.23
KDPI (median of %)^b^	80.0 (49.5; 91.0)	82.5 (61.7; 91.7)	79.0 (54.0; 89.0)	0.003
Mismatches HLA ABDR	2,0 (2,0; 3,0)	2,0 (2,0; 3,0)	2,0 (1,0; 3,0)	0.30
CIT, hours	23.1 (19.4; 28.1)	24.7 (21.5; 31.2)	22.0 (18.5; 27.5)	0.001
DGF, N (%)	187 (51.5)	108 (61.0)	79 (42.5)	< 0.001
21-day WBC count, cell/mm^3^	7000 (5300; 8600)	6900 (5250; 8850)	7050 (5475; 8500)	0.99

ADPKD, autosomal dominant polycystic kidney disease; CIT, cold ischemia time; CKD, chronic kidney disease; DGF, delayed graft function; HLA, human leukocyte antigen; KPDI, kidney profile donor index; PCR, polymerase chain reaction; PRA, panel reactive antibodies.

^a^Missing data = 47.

^b^KDPI is applicable only for deceased donors.

The demographic data were compared between the era (Table 1). There is no difference in the recipients age, however in the antigenemia era, they were less frequently white (46.9 vs. 62.4%, p = 0.007), with longer length in dialysis before transplantation (56.0 vs. 42.0 months, p = 0.03), and with higher frequency of class I cPRA > 80% (13.6 vs. 5.4% p = 0.01). In this group, donors were older (53.0 vs. 52.0 years, p = 0.003), with higher KDPI (82.5 vs. 79.0 medians of %, p = 0.003), and the cold ischemia time was longer (24.7 vs. 22.0 h, p = 0.001). Consequently, the frequency of DGF was higher in the first era (61.0 vs. 42.5%, p < 0.001).

### Immunosuppression during 1-year follow-up

**Figure 2:**
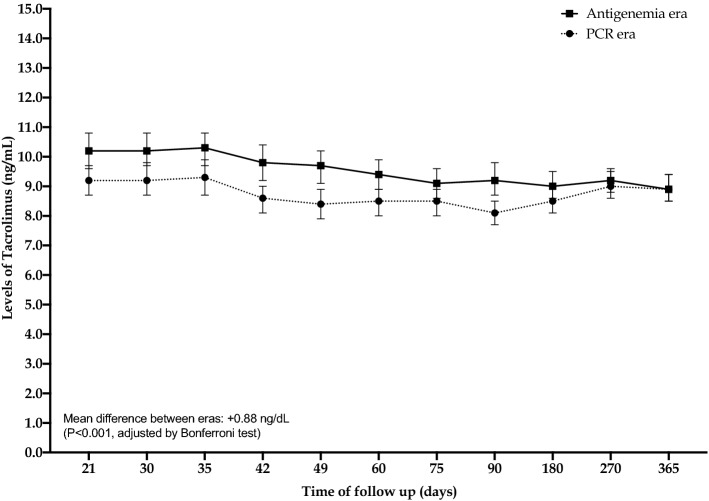
Levels of tacrolimus according to groups (eras) in the first year of transplantation. The mean of eGFR between eras (antigenemia and RT-PCR) was compared by generalized estimating equation modeling and adjusted by Bonferroni test. Squares and circles in the plot represent mean and bars the 95% confidence interval. PCR, real-time polymerase chain reaction.

Over the first year, there was a significant difference in the tacrolimus levels between eras in the mean values in three time-points (antigenemia and PCR, respectively; values expressed in ng/dL and 95% CI in the brackets): 9.8 [9.2–10.4] vs. 8.6 [8.1–9.0] in the day 42; 9.7 [9.1–10.2] vs. 8.4 [7.9–8.9] in the day 49; and 9.2 [8.7–9.8] vs. 8.1 [7.7–8.5]. The overall mean difference in the levels was 0.88 ng/dL higher in the first era (p < 0.001, adjusted by Bonferroni test), and the plot summarizing the evolution over the first year after transplantation is shown in the Fig. 2. The doses of mycophenolate were also compared at different time points in both eras, and no differences were observed (Table 2).

**Table 2:** Doses of acid mycophenolate over the follow-up.

Time after transplantation (days)	Antigenemia era (N = 177)				RT-PCR era (N = 186)				p
	Mycophenolate dose, N (%)				Mycophenolate dose, N (%)				
	1440 mg	1080 mg	720 mg	Withdrawn	1440 mg	1080 mg	720 mg	Withdrawn	
21	148 (83.6)	2 (1.1)	25 (14.1)	2 (1.1)	163 (87.6)	3 (1.6)	15 (8.1)	5(2.7)	0.21
30	144 (81.4)	2 (1.1)	29 (16.4)	2 (1.1)	158 (84.9)	3 (1.6)	18 (9.7)	7 (3.8)	0.11
35	135 (76.3)	2 (1.1)	36 (20.3)	4(2.3)	152 (81.7)	1 (0.5)	28 (15.1)	5 (2.7)	0.53
42	129 (72.9)	3 (1.7)	38 (21.5)	7 (4.0)	135 (72.6)	2 (1.1)	38 (20.4)	11 (5.9)	0.80
49	113 (63.8)	3 (1.7)	50 (28.2)	11 (6.2)	133 (71.5)	3 (1.6)	40 (21.5)	10 (5.4)	0.48
60	110 (62.1)	1 (0.6)	58 (32.8)	8 (4.5)	130 (69.9)	2 (1.1)	44 (23.7)	9 (4.8)	0.31
75	100 (56.5)	2 (1.1)	64 (36.2)	10 (5.6)	121 (65.1)	4 (2.2)	51 (27.4)	8 (4.3)	0.36
90	98 (55.4)	2 (1.1)	71 (40,1)	5 (2.8)	121 (65.1)	6 (3.2)	52 (28.0)	7 (3.8)	0.06
180	75 (42.4)	6 (3.4)	87 (49.2)	7 (4.0)	94 (50.5)	7 (3.8)	73 (39.2)	12 (6.5)	0.15
270	76 (42.9)	7 (4.0)	82 (46,3)	10 (5.6)	90 (48.4)	15 (8.1)	67 (36.0)	14 (7.5)	0.08
365	71 (40.1)	6 (3.4)	88 (49.7)	10 (5.6)	89 (47.8)	15 (8.1)	68 (36.6)	14 (7.5)	0.02

Values expressed in absolute values (%). The missing values are those who used Mycophenolate 360 mg.

### Outcomes

One hundred and seventy-two patients (47.4%) required treatment for CMV due to infection or disease, 47.0 days after transplantation; 79 presented symptoms or laboratory changes attributable to CMV disease (21.8% of the whole population and 45.9% of CMV diagnosed patients). The most common symptom was diarrhea (n = 40), whereas the most common laboratory change was leukopenia (n = 34). Symptoms and laboratory changes at the moment of CMV disease are detailed in supplementary Table 1. Only one patient had an invasive disease. The length of treatment was 22.0 days.

**Figure 3:**
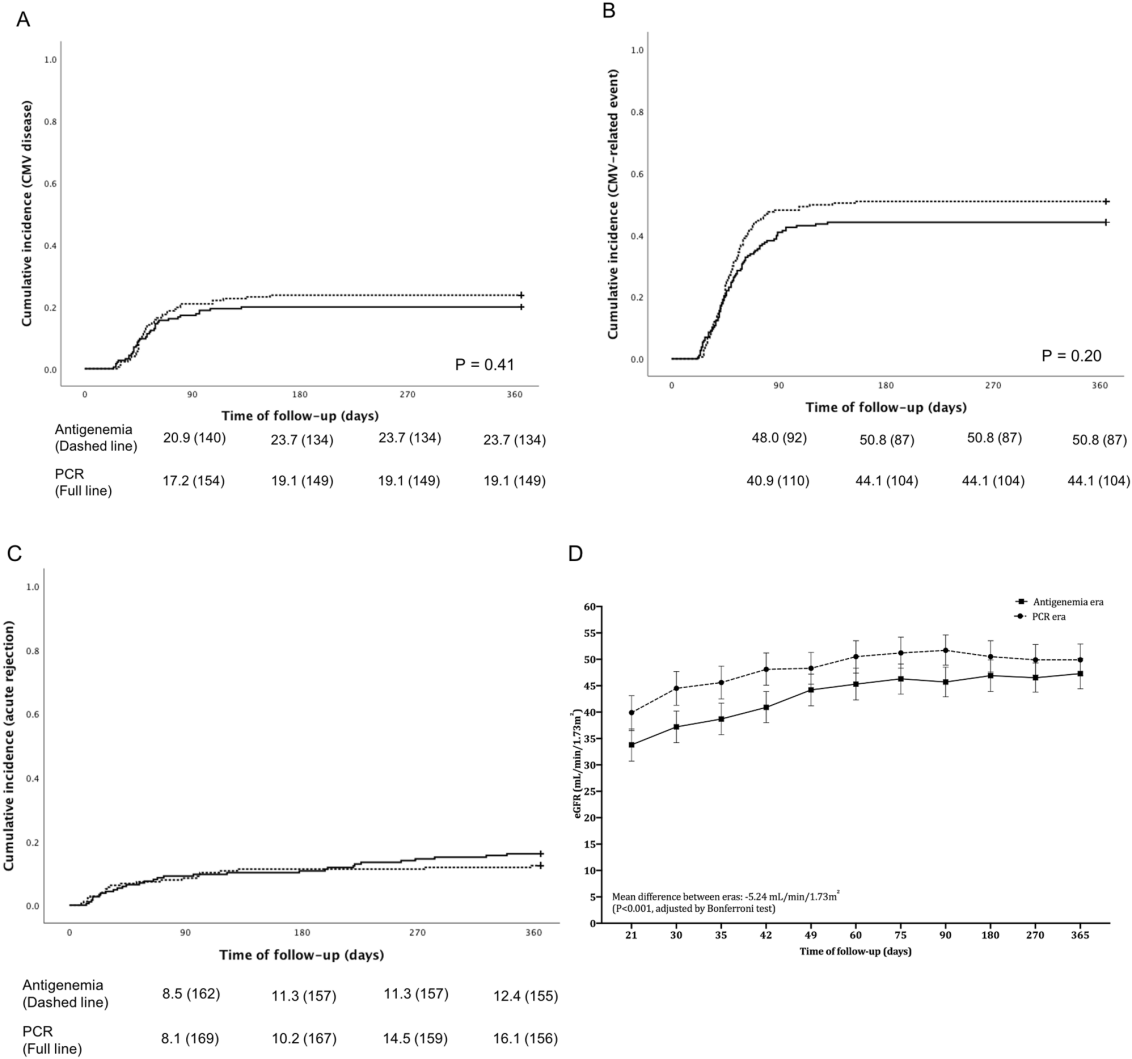
Primary and secondary outcomes: CMV-related events, CMV disease, acute rejection and graft function. (**A**) cumulative incidence of the first CMV-related event (infection or disease) according to the era. (**B**) cumulative incidence of CMV disease according to the era. (**C**) cumulative incidence of acute rejection according to the era. (**D**) graft function assessed by estimated glomerular filtration rate according to the era. In the figures (**A**–**C**), the P-value was calculated by log-rank. The means of graft function over time and according to era were compared by generalized estimating equation modeling and adjusted by Bonferroni test. Squares and circles in the plot represent mean and bars the 95% confidence interval. RT-PCR, real-time polymerase chain reaction].

One-year cumulative incidence of CMV disease was not different according to the era: 23.7% in the antigenemia era vs. 19.1% in the RT-PCR, p = 0.41 (Fig. 3A). In addition, there was no difference in the cumulative incidence of the first CMV-related event: 50.8% in the antigenemia era vs. 44.1% in the PCR, p = 0.20 (Fig. 3B). The time between transplantation and the first event was not different too: 47.0 (37.5; 60.2) vs. 47.0 (36.7; 64.5) days, respectively, p = 0.93. In the first era, the median antigenemia when the treatment was started was 18 cells, and 20 patients (21.5%) had to be treated with less than 10 cells. In the second era, the viral load when the treatment was started was 7,093 IU/mL (5247; 12,327), while 19 patients (22.4%) had to be treated with less than 5000 IU/mL. Furthermore, the length of treatment was longer in the RT-PCR (Table 2): 20.0 vs. 27.5 days, p < 0.001. Last, there was no difference in the requirement for retreatments: 14.7% vs. 11.8%, respectively, p = 0.48.

One-year cumulative incidence of acute rejection (Fig. 3C) was 12.4% in the antigenemia era and 16.1% in the RT-PCR (p = 0.35). In total, 18 patients had acute rejection before CMV-related events. The time between AR and CMV diagnose was 27.5 (15.0; 36.7) days. Figure 3D shows the eGFR over the follow-up time according to both eras. Owing to the difference in the DGF incidence, the eGFR was lower in the first era from the baseline (day 21) to day 42 (antigenemia and RT-PCR, respectively; values expressed in mL/min/1.73 m^2^; 95% CI in the brackets): baseline 33.8 [30.7–36.8] vs. 39.9 [36.6–43.1]; day 42 40.9 [38.0–43.9] vs. 48.1 [45.1–51.2]. The overall mean difference in the graft function was 5.24 mL/min/1.73 m^2^ lower in the first era (p < 0.001, adjusted by Bonferroni test).

### Variables associated with CMV first event and CMV disease

In the Table 3 is shown the univariable and multivariable models for CMV disease (model 1) and first CMV-related event (infection or disease, model 2). The variables were selected in bivariate analysis comparison between patients who had CMV-disease with those who had not (Supplementary Table 2). The same analysis was performed to CMV-related events (Supplementary Table 3).

**Table 3:** Univariable and multivariable analyses for CMV-disease and first CMV-associated event.

Variables	Univariable			Multivariable		
	HR	95% CI	p	HR	95% CI	p
**CMV disease: model 1**						
Era (RT-PCR vs. AgCMV)	0.83	0.53–1.29	0.41			
DM as CKD etiology (yes vs. no)	0.55	0.25–1.20	0.13			
Donor age (each year old)	1.02	1.00–1.03	0.05			
DGF (yes vs. no)	1.99	1.25–3.17	0.004			
21-day WBC count (per each cell/mm^2^)	1.00	1.00–1.00	0.05			
AR within 30 days (yes vs. no)	2.67	1.29–5.56	0.008	2.34	1.12–4.89	0.024
30-day eGFR (each 1 mL/min/1.73 m^2^)	0.98	0.97–0.99	0.001	0.98	0.97–0.99	0.001
**CMV infection or disease (first event): model 2**						
Era (RT-PCR vs. AgCMV)	0.82	0.61–1.11	0.20			
Recipient age (each year old)	1.01	1.00–1.02	0.08			
Hemodialysis (yes vs. no)^a^	0.68	0.39–1.18	0.17			
Retransplant (yes vs. no)	0.62	0.37–1.04	0.07			
Donor age (each year old)	1.01	1.00–1.02	0.06			
DGF (yes vs. no)	1.61	1.18–2.18	0.002			
AR within 30 days (yes vs. no)	2.30	1.33–3.98	0.003	2.05	1.18–3.56	0.01
30-day eGFR (each 1 mL/min/1.73 m^2^)	0.98	0.97–0.99	< 0.001	0.98	0.97–0.99	< 0.001

Variables included in the model 1: era (antigenemia or RT-PCR), diabetes as CKD etiology, donor age, DGF, 21-day WBC count, 30-day eGFR and AR within 30 days after transplantation. KDPI was excluded due to collinearity with donor age. The model’s AUC-ROC to predict CMV disease: 0.743 (95% IC 0.681–0.806). Variables included in the model 2: era (antigenemia or RT-PCR), recipient age, hemodialysis as replacement renal therapy before the transplantation, retransplant, donor age, DGF, 30-day eGFR and AR within 30 days after transplantation. Cold ischemia time was excluded due to collinearity with DGF.

The accuracy of the multivariable modeling for predicting CMV-related event was assessed by an AUC-ROC, which achieved a result of 0.792 (95% CI 0.745–0.839). AR, acute rejection; CKD, chronic kidney disease; DGF, delayed graft function; eGFR, estimated glomerular filtration rate; WBC, white blood cells.

^a^Hemodialysis as the renal replacement therapy before transplantation].

In the model 1, acute rejection within 30 days was associated with a twofold increased risk of CMV disease (HR yes vs. no = 2.34; 95% CI = 1.12–4.89; p = 0.02), while each 1 mL/min/1.73 m^2^ increase of 30-day eGFR was associated with a 2% reduced risk (HR for each 1 mL/min/1.73 m^2^ = 0.98; 95% CI = 0.97–0.99; p = 0.001). This model achieved an AUC-ROC of 0.743 (95% CI = 0.681–0.806). Yet, in the model 2, the same variables were associated with the probability of first CMV-related event (infection or disease, which occurs first): AR within 30 days (HR yes vs. no = 2.05; 95% CI = 1.18–3.56; p = 0.01) and 30-day eGFR (HR for each 1 mL/min/1.73 m^2^ = 0.98; 95% CI 0.97–0.99; p < 0.001).The AUC-ROC for the model 2 was of 0.792 (95% CI 0.745–0.839).

## Discussion

Despite improved kidney transplantation clinical management, the CMV infection is still a concern^4^. According to the best clinical guidelines, both strategies available for preventing the consequences of CMV infection, universal prophylaxis or preemptive treatment, present advantages and some disadvantages, and centers should opt for one or another, considering their characteristics^4^. For example, in Brazil, more than 90% of kidney transplantation is supported by the public health system, and the costs of universal prophylaxis are not disbursed, which can occur in other low and mid-income countries^11^. In the present study, we compared the main CMV-related outcomes when we transitioned from pp65 to RT-PCR in kidney transplant recipients receiving preemptive treatment as a strategy to prevent CMV infection and identified potential clinical predictors of the CMV-related events.

In our primary hypothesis, we were expecting to detect a reduction in the rate of symptomatic patients, considering that the RT-PCR has high sensitivity to detect low viral load^15,16^. Moreover, antigenemia presents several limitations in CMV treatment, highlighted in the updated international consensus in 2013 as the lack of standardization, the dependence of the subjective interpretation, and its performance when the count of neutrophils is low^14^. Consequently, since that, quantitative nucleic acid amplification testing has been established as the "cornerstone for diagnosis and monitoring for CMV infection and disease"^4,14^. Indeed, using a threshold of 5000 IU/mL to start the preemptive treatment in the RT-PCR era, the frequency of treatment in our cohort was not different from those observed with pp65 antigenemia, and the time to treatment onset was precisely the same. Additionally, we did not find the expected reduction in the rate of patients who had CMV disease.

When the treatment is started, the viral load seems to be associated with the clinical resolution^17^. In an exploratory analysis from the VICTOR study, where plasma samples of 267 participants were retested, and the viral load was calibrated based on the CMV World Health Organization, the faster resolution of CMV disease after treatment with valganciclovir was 57% more likely when the initial viral load was lower than 18,200 IU/mL^17^. Different from the VICTOR study, in our cohort, all patients were conducted under the preemptive treatment with closer viral load screening; therefore, the main target of the clinical management was to avoid the symptomatic infection. After that, the median of viral load was 7093 IU/mL, and 75% of patients had a viral load lower than 12,327 IU/mL. Last, despite the low frequency of invasive disease, we consider that the rate of symptomatic infection was higher than we expected when we transitioned from antigenemia to RT-PCR.

Although the local clinical approach in the first era had preconized 1-week extension in the treatment after the last negative antigenemia, the duration of treatment to reach a viral load suppression was longer with RT-PCR. There was an initial concern that highly sensitive assays to manage the CMV infection resulted in prolonged treatments and unnecessary exposure to antiviral therapy^14^. However, a shorter time of treatment using standardized quantitative nucleic acid testing has been demonstrated in a previous study^17,18^. Additionally, as a direct consequence of a more prolonged treatment time with RT-PCR observed in our study, we expected that the rate of the retreating requirement was reduced, considering that reaching a virological suppression seems to be predictive of clinical response^17^. Indeed, the need for retreatment was slightly lower in the PCR era (11.8% vs. 14.7%); however, this difference was not significant. Taking these results together and the indirect evidence figured in the present study, it is possible to speculate that a cut-off lower than 5000 IU/mL could reach lower rates of symptomatic patients than we observed. On the other hand, it seems to be that a cut-off of 10 positive cells associated with a seven-day extended treatment would be equivalent to the viral load suppression achieved by treatment guided for PCR, and this find can be helpful for centers that have only antigenemia as the option to conduct the preemptive treatment.

In a secondary analysis, we sought predictors of CMV-related events in patients receiving preemptive treatment. More recently, the quantification of the T-cell-specific response against CMV antigens has been considered a promisor tool for predicting the CMV-related events^19^, and it would be helpful for preemptive treatment optimization^20^. However, its use is not standardized for wieldy clinical use. Here, two clinical predictors were associated with the probability of the first CMV-related event: early acute rejection and 30-day graft function.

The association between acute rejection and CMV replication is mainly supported by immunosuppression intensification to treat the immunological event^21^. Therefore, we included the acute rejection within 30 days as an independent variable in the multivariable model. Of note, early acute rejection was also an independent predictor for CMV disease, which supports the correlation between the intensity of immunodepression and the spectrum of infection. Furthermore, the association of early graft function and CMV-related events has been previously reported^22^, and was confirmed by our group in an independent cohort of 938 patients transplanted between 2014 and 2015, where reduced 30-day eGFR was a strong predictor of CMV infection or disease: the odds ratio (OR) for each 1 mL/min/1.73 m^2^ was 0.98; 95% CI 0.97–0.99^23^. In this new cohort, we confirmed this observation in the Cox regression analysis, where every 1 mL/min/1.73 m^2^ increase of 30-day eGFR was associated with a 2% reduced risk of CMV-related events. The pathophysiological processes involved in this association are still unknown. Reduced graft function at 30 days might be the consequence of persistent ischemia and reperfusion injury and release of cytokines such as TNFα, IL-6, and IL-1β that are associated with increased risk of CMV replication^24^. Conversely, reduced kidney function may influence the pharmacokinetics of immunosuppressive drugs such as mycophenolate acid, possibly increasing the net state of immunosuppression^25^.

Our study has several limitations. First, a historical study, carried out in a single-center, with groups followed in two different eras, is associated with some biases. Yet, some exclusion criteria limit the extrapolation of the results, such as IL2-RA induction treatment, the D+R− serostatus patients, and the use of mTORi as maintenance immunosuppression. Second, in both periods, the thresholds for starting the preemptive treatment were defined by the clinical routines due to the lack of robust evidence to support a prespecified cut-off. Third, some differences in the baseline characteristics were observed when both eras were compared, mainly in the donor's demography, which could be associated with worse graft function 30 days after transplantation, although the model to evaluate predictors of CMV-related events has been adjusted for eras. Last, the adherence to the local approach was not directly measured.

In conclusion, in the present study, we did not observe a reduction in the frequency and in the time for CMV-related events, as well as in the requirement for retreatments when the antigenemia was transitioned to quantitative nucleic acid amplification testing, using a threshold of 5000 IU/mL in the standardization RT-PCR for starting the preemptive treatment in kidney transplant recipients. These data also support using RT-PCR or traditional CMV pp65 antigen for the preemptive management, which could be helpful in most centers that have no easy access to RT-PCR. Finally, we defined 30-day graft function and early acute rejection as clinical predictors of CMV replication after transplantation.

## Methods

### Study design and population

This was a retrospective sequential single-center cohort study carried out at Hospital do Rim–São Paulo, Brazil. Considering that the study was aimed to evaluate a transition in methods chosen to assess CMV viremia in the preemptive treatment (antigenemia or PCR), patients were grouped in two different eras: the use of antigenemia in the first era and the use of RT-PCR in the second one. The study was conducted following the Declaration of Helsinki and was approved by the Ethics Committee at Federal University of São Paulo (identification number CAEE 05677618.6.0000.5505, and approval number 3.164.538). Being a retrospective study, the informed consent form was waived by the Ethics Committee at Federal University of São Paulo.

The eligible participants were CMV seropositive kidney transplant recipients who underwent kidney transplants between March 2016 and August 2018, under preemptive treatment for the risk reduction of CMV disease, and who completed 1 year of follow-up. Other inclusion criteria were: age at the transplantation time older than 18 years, immunological induction with thymoglobulin, and the immunosuppression regime of maintenance based on tacrolimus and mycophenolate, owing to it was the main indication for the preemptive treatment according to the local approach. According to the local protocol to manage the risk of CMV-related events, patients receiving mTOR or azathioprine as a maintenance immunosuppression regimen are not followed by the preemptive treatment; therefore, they were excluded. In addition, recipients of kidney transplants combined with another solid organ or negative CMV serology were excluded. The time for transition from antigenemia to RT-PCR was from March 2017 to February 2018, when patients started treatment based on antigenemia, but the antiviral was usually interrupted based on the result of PCR. Therefore, patients transplanted in this period were excluded too.

### Immunosuppression and prophylaxis

All patients received a single dose of 3.0 mg/kg of Thymoglobulin as an induction strategy, following the local practice, which was previously published^26–28^. The maintenance immunosuppression regime consisted of a combination of tacrolimus, prednisone, and acid mycophenolate. The initial dose of tacrolimus was 0.1 mg/kg BID for recipients with a panel reactive antibodies (cPRA) ≥ 50%. For those with PRA < 50%, the same dose was started in recipients of extended criteria deceased donors, while 0.05 mg/kg BID was indicated for recipients of living donors or standard criteria deceased donors. Regarding mycophenolate, the initial dose was 720 mg BID, adjusted in the presence of attributable side effects. The dose of tacrolimus was adjusted to maintain C_0_ levels between 5 and 15 ng/mL. In addition, all patients underwent prophylaxis with albendazole for parasitic infections and sulfamethoxazole-trimethoprim for *Pneumocystis jirovecii*.

### Monitoring and treatment of CMV infection

For the preemptive treatment, viremia was collected every 2 weeks from the 21st after transplantation. The CMV tests results were available 1 day after sample collection. When patients presented the preemptive treatment criteria, the antiviral was started 2 or 3 days after the sample collection. For pp65 antigenemia, after peripheral blood extraction, leukocytes were incubated with C10/C11 antibodies and other reagents from the CMV Brite Turbo kit (IQ^®^ Products, Groningen, Netherlands). The presence of pp65 antigen was detected by a homogeneous yellow-green nuclear pattern in a fluorescence microscope, and the final result was expressed by the number of positive cells per 200,000 leukocytes^29^. The RT-PCR was performed with a commercial Abbott RealTime CMV kit. The DNA extraction, amplification, and detection were performed in the automated Real-Time m2000 system (Abbott Molecular Inc), having the DNA sequences of the UL34 and UL80.5 CMV genes as targets^15^. The procedure consisted of a real-time amplification reaction on a microplate, with programmable temperature control and variation, and simultaneously an optical fluorescence detection system with the reaction in a thermocycler^16,30^. The reported limits of detection and quantification were 31.2 IU/mL.

In the antigenemia era, the preemptive treatment was indicated in the presence of 10 or more positive cells in asymptomatic patients or in patients who presented symptoms attributable to CMV infection, independent of the number of positive cells. In the RT-PCR era, the preemptive treatment was indicated in the presence of 5000 IU/mL or more in asymptomatic patients or in patients who presented symptoms attributable to CMV infection, independent of viral load in the RT-PCR. The treatment consisted of intravenous ganciclovir 5 mg/kg twice a day, adjusted for renal function. During treatment, monitoring was carried out weekly. For the antigenemia era, the treatment was extended for seven following days from the first negative result. On the other hand, in the RT-PCR era, the treatment was interrupted when the result was undetectable (< 31 IU/mL)^31^. Monitoring after the treatment interruption was maintained over the following 3 months. For patients with CMV-disease (symptomatic patients), but with a low viral load, the minimal length time of treatment was 3 weeks. In cases of recurrence, the retreatment criteria, including the time for antiviral treatment interruption, were the same described above for each era.

### Definitions

Cytomegalovirus infection was classified according to the Third International Cytomegalovirus Consensus as CMV infection, CMV disease, and invasive disease^4^: infection was defined by the evidence of viral replication in the absence of symptoms attributable to the viral activity, whereas disease was determined by evidence of CMV replication, associated with attributable symptoms or laboratory abnormalities, and the invasive disease was defined by the presence of the virus in the histological analysis of any tissue regardless of the result of the viremia or by retinitis, meningitis, or encephalitis. Recurrences of CMV infection or disease were defined by the need for a new treatment after the complete remission of the previous episode. Delayed graft function was defined by the need for dialysis during the first week, and acute rejection (AR) as treated rejections, proven by biopsy or not, according to Banff's classification^32^. The estimated glomerular filtration rate (eGFR) was calculated by the CKD-EPI equation^33^.

### Outcomes

The primary outcome was a composed of CMV disease and invasive-CMV disease, which occurs first.

CMV-related event defined as infection or CMV disease. The incidence of disease, the time for detecting events, the length of treatment with ganciclovir, and the frequency of retreatment requirement were compared according to the era. The incidence of CMV-related events, considering infection (only asymptomatic patients) and CMV-disease, which occurs first, the incidence of acute rejection and 1-year graft function were secondarily evaluated.

### Statistical analysis

Continuous variables are summarized as the median and interquartile range (1st; 3rd) and compared by test U of Mann–Whitney, and categorical variables are summarized as absolute and relative frequencies and compared by the *X*^*2*^ test or Fisher's exact test. These comparisons were fitted for the era (antigenemia vs. RT-PCR).

The cumulative incidence of CMV-related events, CMV disease, and acute rejection were calculated by Kaplan–Meier and compared by log-rank test. The frequency of retreatment requirement was compared by the *X*^2^ test. Time for detecting CMV-related events and the length of treatment with ganciclovir according to era were compared by test U of Mann–Whitney. For graft function, a generalized estimated equation was performed to compare the mean of eGFR between eras (antigenemia and RT-PCR). The model was adjusted by the Bonferroni test. The same approach was performed to compare tacrolimus levels between eras.

The potential clinical predictors for the primary outcomes (CMV-related events and CMV disease) were analyzed by the proportional hazard ratios (HR) throughout the Cox regression modeling (backward stepwise). The variables for the model were selected in bivariable analyses comparisons of patients who had CMV-related events with those who did not (supplementary material). The same approach was performed to select candidates variables related to CMV disease. Variables that reached a P-value < 0.20 were considered for the final modeling. The median was imputed for the only variable with missing values, 30-day eGFR (1.38%). The accuracy of the final model was assessed by the area under a receiver operating characteristic (AUC-ROC). Statistical analyses were performed using Statistical Package for the Social Sciences (version 26; IBM, Armonk, NY, USA), and statistical significance was defined as p < 0.05, with the 95% confidence interval.

## Supplementary Information

Supplementary Tables.

## Data Availability

The datasets used and/or analyzed during the current study are available on reasonable request from the corresponding author, who can be contacted at lucio.requiao@gmail.com.

## Abbreviations

ADPKD: Autosomal dominant polycystic kidney disease
AR: Acute rejection
CI: Confidence interval
CIT: Cold ischemia time
CKD: Chronic kidney disease
CMV: Cytomegalovirus
cPRA: Calculated panel reactive antibody
CTS: Collaborative transplant study
DGF: Delayed graft function
DNA: Deoxyribonucleic acid
eGFR: Estimated glomerular filtration rate
GEE: Generalized estimating equation
GLMM: Generalized linear mixed models
HLA: Human leukocyte antigen
HR: Hazard ratio
KDPI: Kidney donor profile index
mTOR: Mammalian target of rapamycin
RT-PCR: Real-time polymerase chain reaction
