# Supplementary material for: Transition from antigenemia to quantitative nucleic acid amplification testing in cytomegalovirus-seropositive kidney transplant recipients receiving preemptive therapy for cytomegalovirus infection
Source: Sci Rep. 2022 Jul 27;12:12783. doi: 10.1038/s41598-022-16847-3 (PMC9329426; doi:10.1038/s41598-022-16847-3)
Supplement: Supplementary file 1 — Supplementary Tables. [file 41598_2022_16847_MOESM1_ESM.docx]

**Title:**

Transition from antigenemia to quantitative nucleic acid amplification testing in cytomegalovirus-seropositive kidney transplant recipients receiving preemptive therapy for cytomegalovirus infection.

**Authors:**

Mônica Rika Nakamura^1,2^, Lúcio R. Requião-Moura*^1,2^, Roberto Mayer Gallo^1^, Camila Botelho^1^, Júlia Taddeo^1^, Laila Almeida Viana^1^, Cláudia Rosso Felipe^2^, José Medina-Pestana^1,2^, Hélio Tedesco-Silva^1,2^.

**Affiliations:**

1. Kidney Transplantation Division, Hospital do Rim, Fundação Oswaldo Ramos, São Paulo – Brazil.
2. Nephrology Division, Department of Medicine, Universidade Federal de São Paulo, São Paulo – Brazil.

***Supplementary Table 1. Symptoms and laboratorial changings attributable to CMV disease.***

| **Event** | **Total**  **(N=79)** | **Antigenemia**  **Era (N=42)** | **PCR Era**  **(N=37)** |
| --- | --- | --- | --- |
| **Diarrhea, N** | 40 | 18 | 22 |
| **Leukopenia, N** | 34 | 15 | 19 |
| **Nausea/ Vomiting, N** | 8 | 5 | 3 |
| **Abdominal Pain, N** | 7 | 6 | 1 |
| **Thrombocytopenia, N** | 7 | 2 | 5 |
| **Anemia, N** | 6 | 2 | 4 |
| **Fatigue, N** | 6 | 2 | 4 |
| **Inappetence, N** | 4 | 2 | 2 |
| **Fever, N** | 3 | 2 | 1 |
| **Malaise, N** | 3 | 2 | 1 |
| **Melena*, N** | 1 | 0 | 1 |
| **Pneumonia^#^, N** | 2 | 1 | 1 |
| **Invasive disease, N** | 1 | 1 | 0 |

[*In the patient with melena, colonic ulcers were found in the colonoscopy; however, the CMV was not identified in the pathology; ^#^In one patient with pneumonia, bronchoscopy, bronchoalveolar lavage, and lung biopsy did not confirm the invasive infection. The final diagnosis was infection by *Mycobacterium tuberculosis*. For the other one, no invasive method was performed.]

***Supplementary Table 2. Comparison of patients who had CMV disease with those who had not.***

| **Variables** | **CMV disease** | | **P** |
| --- | --- | --- | --- |
|  | **Yes** | **No** |  |
| **Recipient age (years)** | 49.0 (40.0; 56.0) | 49.0 (37.0; 58.0) | 0.79 |
| **Recipient sex – male, n (%)** | 40 (50.6) | 159 (56.0) | 0.40 |
| **Recipient white, n (%)** | 46 (58.2) | 153 (53.9) | 0.49 |
| **Diabetes as cause of CKD, n (%)** | 7 (8.9) | 45 (15.8) | 0.12 |
| **Time on dialysis (months)** | 51.0 (18.0; 84.0) | 41.5 (22.0; 75.5) | 0.77 |
| **Hemodialysis, n (%)** | 73 (92.4) | 267 (94.0) | 0.60 |
| **Retransplant, n (%)** | 8 (10.1) | 38 (13.4) | 0.44 |
| **PRA Class I (medians of %)** | 0.00 (0.00; 30.0) | 0.00 (0.00; 28.0) | 0.67 |
| **PRA Class II (medians of %)** | 0.00 (0.00; 0.00) | 0.00 (0.00; 0.00) | 0.75 |
| **Deceased donor, n (%)** | 77 (97.5) | 272 (95.8) | 0.49 |
| **Donor age (years)** | 54.0 (49.0; 60.0) | 52.0 (40.0; 60.0) | 0.07 |
| **Donor sex – male, n (%)** | 39 (49.4) | 153 (53.9) | 0.48 |
| **Donor white, n (%)** | 37 (46.8) | 152 (53.5) | 0.29 |
| **Donor CMV-IgG, *positive*, N (%)*** | 62 (93.9) | 228 (91.2) | 0.62 |
| **KDPI (medians of %)**** | 84.0 (66.0; 91.0) | 77.0 (47.0; 91.0) | 0.07 |
| **Mismatches HLA ABDR (number)** | 2.0 (2.0; 3.0) | 2.0 (1.0; 3.0) | 0.23 |
| **CIT (hours)** | 22.7 (19.5; 28.2) | 23.6 (19.9; 28.5) | 0.97 |
| **DGF, n (%)** | 52 (65.8) | 135 (47.5) | 0.004 |
| **21-day WBC count (cells/mm^3^)** | 5,900 (4,800; 7,900) | 7,150 (5,800; 8,800) | 0.005 |
| **30-day tacrolimus level (ng/dL)** | 8.9 (6.7; 12.0) | 9.1 (7.1; 11.8) | 0.70 |
| **30-day eGFR (mL/min/1.73m^2^)** | 30.8 (20.8; 41.1) | 38.7 (28.1; 56.5) | <0.001 |
| **AR within 30 days, n (%)** | 8 (10.1) | 10 (3.5) | 0.02 |

[AR, acute rejection; CIT, cold ischemia time; DGF, delayed graft function; eGFR, estimated glomerular filtration rate; HLA, human leukocyte antigen; KPDI, kidney profile donor index.

* Missing data = 47

**KDPI is applicable only for deceased donors. 30-day represents 30 days after transplantation.]

***Supplementary table 3. Comparison of patients who had CMV-related event with those who had not.***

| **Variables** | **CMV-related event** | | **P** |
| --- | --- | --- | --- |
|  | **Yes** | **No** |  |
| **Recipient age (years)** | 50.0 (40.0; 58.0) | 48.0 (36.0; 57.0) | 0.14 |
| **Recipient sex – male, n (%)** | 91 (52.9) | 108 (56.5) | 0.49 |
| **Recipient white, n (%)** | 95 (55.2) | 104 (54.5) | 0.88 |
| **Diabetes as cause of CKD, n (%)** | 23 (13.4) | 29 (15.2) | 0.62 |
| **Time on dialysis (months)** | 47.0 (23.0; 81.0) | 37.0 (19.5; 72.0) | 0.26 |
| **Hemodialysis, n (%)** | 158 (91.9) | 182 (95.3) | 0.18 |
| **Retransplant, n (%)** | 16 (9.3) | 30 (15.7) | 0.07 |
| **PRA Class I (medians of %)** | 0.00 (0.0; 22.0) | 0.0 (0.0; 43.5) | 0.61 |
| **PRA Class II (medians of %)** | 0.0 (0.0; 0.0) | 0.0 (0.0; 0.0) | 0.76 |
| **Deceased donor, n (%)** | 165 (95.5) | 184 (96.3) | 0.84 |
| **Donor age (years)** | 54.0 (44.0; 61.0) | 51.0 (38.0; 59.0) | 0.03 |
| **Donor sex – male, n (%)** | 90 (52.3) | 102 (53.4) | 0.84 |
| **Donor white, n (%)** | 95 (55.2) | 104 (54.5) | 0.88 |
| **Donor CMV-IgG, *positive*, N (%)*** | 135 (93.8) | 155 (90.1) | 0.24 |
| **KDPI (medians of %)**** | 84.0 (61.0; 92.0) | 74.5 (46.0; 90.0) | 0.61 |
| **Mismatches HLA ABDR (number)** | 2.0 (2.0; 3.0) | 2.0 (1.0; 3.0) | 0.76 |
| **CIT (hours)** | 23.1 (19.4; 27. 8) | 24.0 (20.4; 28.8) | 0.01 |
| **DGF, n (%)** | 102 (59.3) | 85 (44.5) | 0.005 |
| **21-day WBC count (cells/mm^3^)** | 6,700 (5,225; 8,700) | 7,100 (5,800; 8,600) | 0.31 |
| **30-day tacrolimus level (ng/dL)** | 9.1 (7.1; 11.8) | 9.1 (7.1; 12.0) | 0.77 |
| **30-day eGFR (mL/min/1.73m^2^)** | 32.5 (21.9; 44.5) | 42.0 (29.7; 59.5) | <0.001 |
| **AR within 30 days** | 14 (8.1) | 4 (2.1) | 0.008 |

[AR, acute rejection; CIT, cold ischemia time; DGF, delayed graft function; eGFR, estimated glomerular filtration rate; HLA, human leukocyte antigen; KPDI, kidney profile donor index.

* Missing data = 47

**KDPI is applicable only for deceased donors. 30-day represents 30 days after transplantation.]
